# The Effects of the COVID19-Related Lockdown Are Modulated by Age: An Italian Study in Toddlers and Pre-Schoolers

**DOI:** 10.3390/brainsci11081051

**Published:** 2021-08-09

**Authors:** Mariangela Cerasuolo, Serena Malloggi, Francesca Conte, Benedetta Albinni, Oreste De Rosa, Marissa Lynn Rescott, Fiorenza Giganti, Gianluca Ficca

**Affiliations:** 1 Department of Psychology, University of Campania L. Vanvitelli, Viale Ellittico 31, 81100 Caserta, Italy; mariangela.cerasuolo@gmail.com (M.C.); francesca.conte@unicampania.it (F.C.); benedettaalbinni@gmail.com (B.A.); oreste.derosa@unicampania.it (O.D.R.); marissalynn.rescott@unicampania.it (M.L.R.); gianluca.ficca@unicampania.it (G.F.); 2 Department NEUROFARBA, University of Firenze, Via di San Salvi 12, 50135 Firenze, Italy; fiorenza.giganti@unifi.it

**Keywords:** COVID-19 pandemic, children’s sleep schedules, children’s sleep quality

## Abstract

Although the issue has been repeatedly explored, data on the impact of the COVID-19 pandemic on children’s sleep quality are inconsistent. To clarify these discrepancies, here we investigate possible age-related differences. During the lockdown, 112 parents of toddlers (0–3 years, N = 61) and pre-schoolers (4–5 years, *n* = 51) completed an online survey including the Children’s Sleep Habits Questionnaire (CSHQ). Sleep-related items required an additional retrospective judgment, referring to the pre-pandemic period. During the lockdown, sleep schedules were delayed in both age groups whereas sleep quality (CSHQ total scores) improved in pre-schoolers but not in toddlers. Between-groups comparisons revealed that, prior to the lockdown, pre-schoolers showed worse sleep quality than toddlers, whereas this difference disappeared during home confinement. Also, pre-schoolers’ sleep timing was advanced before the lockdown and delayed during the lockdown relative to toddlers’. Our data highlight a significant modulation of age on the impact of the pandemic crisis on sleep, with pre-schoolers experiencing greater effects than toddlers. This profile suggests that factors affecting sleep features have different weights at different ages: sleep patterns would be mainly determined by developmental factors (i.e., biological drive) in younger children, whereas environmental factors (e.g., major lifestyle changes) would have a stronger effect on older ones.

## 1. Introduction

Italy was the first European country to fight against the COVID-19 infection, with the first outbreaks in the North of the country occurring at the end of February 2020. Within a few days, the contagion rapidly spread across the country. To contain the infection, the Italian Government imposed a nationwide lockdown, lasting from 9 March–18 May 2020. During this period, a range of severe disease control strategies were undertaken, such as home confinement, movement restrictions, smart remote working, and temporary closure of non-essential businesses and schools of every order and degree. Individuals were allowed to leave their homes only for demonstrated necessities, such as health reasons, basic needs, and work. Even though these measures proved useful in preventing further spread of the infection and reducing the overload in intensive care units, this prolonged home confinement had a remarkable impact on people’s mental health [1], that may persist in the long-term [2]. In fact, the pandemic emergency represents a complex, unprecedented, psychosocial stressor and the strategies adopted to prevent the contagion have resulted in high rates of anxiety, depression, fear, and panic symptoms [3,4,5]. The lockdown has also markedly disrupted daily social routines and reduced sunlight exposure, with significant consequences on sleep and circadian rhythms. In fact, studies from different countries have shown significant delays in bed- and rise times [6,7,8], worsened sleep quality [7,8,9,10] and increases in insomnia symptoms and sleep disorders [3,4] in adults during the national lockdowns. 

Recently, greater attention has been paid to the effects of the pandemic on pre-school and school-aged children, since the abrupt changes in their daily schedules and sleep-wake patterns, due to extended school closure and social isolation, might represent a risk for both physical and psychological well-being [11]. Studies conducted in several countries during the lockdowns showed that, according to parental report, children’s bed- and rise times were significantly delayed [12,13,14,15,16] and sleep duration was increased [12,13,17,18]. 

However, findings regarding the impact of pandemic-related restrictions on children’s sleep quality are still inconsistent. On one hand, some studies found a worsening of parent-reported sleep quality, as indexed by a sleep disturbances score (in 2-years old toddlers [17], in children aged 6–10 years [15], in children aged 1–12 years old but not in adolescents [16]) and by single Likert-scale items on children’s general sleep quality (in children aged 1–5 years, [18]), bedtime resistance, and freshness at awakening [19]. Another interesting study [20] showed that the worsening of sleep quality in children occurred in the initial, acute phase of the pandemic (April 2020) but disappeared at a two-months follow up.

On the other hand, other studies found either no change during the pandemic (i.e., no increase of sleep disturbances in children aged 2–5 years [14]) or even an improvement of sleep quality (in 4–6 years old pre-schoolers [13]), assessed through the Children’s Sleep Habits Questionnaire (CSHQ, [21]). Zreik et al. [10] also found that most mothers of their sample reported no changes in their children’s sleep quality during home confinement nor any perception of their children’s sleep as being problematic. 

In sum, extant literature clearly points to changes in children’s sleep/wake schedules brought about by the pandemic and related restrictions. Instead, its impact on sleep quality in this population is still poorly understood, possibly due to the age-ranges differences across studies and to the different measures adopted. Therefore, here we aim to investigate whether the pandemic and related lockdown could have differently affected sleep habits and quality according to children’s age, namely in toddlers (0–3 years old) and pre-school children (4–5 years old), considering that, during the first years of life, sleep undergoes important quantitative, qualitative and organizational changes [22,23,24,25]. To this aim, we adopted for both age-groups the same parent-report instrument, the Children’s Sleep Habits Questionnaire (CSHQ, [21]), which allows a detailed assessment of a broad range of sleep dimensions. In addition, we assess parents’ sleep quality and their associations with children’s sleep, in order to better understand this relationship and replicate previous findings on this topic [10,14,15].

## 2. Materials and Methods

### 2.1. Participants and Procedure

An anonymous online survey was administered to Italian parents/caregivers of pre-school children during the Italian lockdown period (from 29 April to 26 May 2020) through the Google Form platform. Considering the unpredictability of changes in restrictive measures (i.e., interruption of the total lockdown), a convenience sample was recruited (through advertisements on research-related websites and social media groups) in order to obtain a sufficient sample size as fast as possible. There was no monetary or credit compensation for participating in the study. The only inclusion criterion was having children aged between 0 and 5 years. A total of 112 parents (mothers = 100; mean age = 36.84 ± 7.26) completed the survey and were included in data analysis. 

After providing informed consent, participants responded to a set of general socio-demographic questions about themselves (e.g., age, gender, education, region of residence, employment, child and other relative’s management) and their children (age, gender) as well as to a few ad-hoc questions related to the COVID-19 emergency. These were followed by a set of standardized questionnaires, i.e., the Pittsburgh Sleep Quality Index (PSQI, [26]) and the Children’s Sleep Habits Questionnaire (CSHQ, [21]), to assess parents and children’s sleep quality, respectively, and the Perceived Stress Scale (PSS-10, [27]), to assess parent’s perception of stress. Importantly, all items regarding sleep required the participant to report both on their current situation and, retrospectively, on the month preceding the lockdown. 

The survey took about 15–20 min to be completed. The study protocol was approved by the ethical committee of the University of Campania “L. Vanvitelli” (code: 14/2020) and was conducted in accordance with the Declaration of Helsinki.

### 2.2. The Survey 

In the first section of the survey, we assessed parents’ perceptions of the COVID-19 emergency, as well as the consequent changes in their daily lives. Participants were asked whether they knew someone who was positive for COVID-19 or deceased because of it, whether their working condition had changed during the lockdown (i.e., “working from home/remote working”, “stopped working”), to what extent their lifestyle was affected (i.e., “not at all”, “a little”, “somewhat”, “much”), how their activity level had changed (i.e., “more sedentary”, “as usual”, “more active”), how afraid they felt (i.e., “not at all”, “moderately”, “extremely”), and an evaluation of their mood (on a 5-point scale from “extremely positive” to “extremely negative”). 

Parents’ stress level was measured through the 10-item version of the Perceived Stress Scale (PSS-10 [27]), which assesses “how unpredictable, uncontrollable, and overloaded” respondents find their lives. Total scores range from 0 to 40, with higher scores indicating higher levels of perceived stress [27]. 

Parents’ sleep quality was assessed through the PSQI [26]. In order to obtain retrospective assessments, all items of this questionnaire required two answers: one referring to the current situation and one to the participant’s situation before the lockdown. The PSQI cut-off score was used to discriminate “good” from “poor” sleepers (scores > 5 indicate poor sleep quality, [28]). Habitual bedtimes (hh:mm) and rise times (hh:mm) were also obtained from the two corresponding items of the PSQI. 

Children’s sleep was assessed through the Children’s Sleep Habits Questionnaire (CSHQ, [21]). The CSHQ is a parent-report questionnaire, widely used to examine sleep behaviour and habits in pre-school [29] and school-aged children [30]. Recently, the CSHQ has also been employed in a few studies to assess sleep characteristics in infants [31,32]. In order to obtain retrospective assessments, parents were asked to respond to each item of the questionnaire both by referring to their children’s current situation and to their situation before the lockdown. The questionnaire provides a total score (CSHQ total score) as well as scores to eight subscales: (1) Bedtime Resistance (BR), (2) Sleep Onset Delay (SOD), (3) Sleep Duration (SD), (4) Sleep Anxiety (SA), (5) Night Waking (NW), (6) Parasomnias (P), (7) Sleep Disordered Breathing (SDB), and (8) Daytime Sleepiness (DS). Items are rated on a 3-point scale ranging from ‘‘usually’’ (five to seven times/week) to ‘‘sometimes’’ (two to four times/week) to ‘‘rarely’’ (zero times to one time/week), with higher scores indicating more disturbed sleep. A total score of 41 has been reported to be a sensitive clinical cut-off for the identification of probable sleep problems [21]. The questionnaire also includes items regarding bed- and rise times during both weekdays and weekends. 

### 2.3. Data Analysis

Statistical analyses were performed using the SPSS statistics software (version 27.0, IBM Corp., Armonk, NY, USA). Due to the non-normal distribution of variables (as assessed through the Shapiro–Wilk test), non-parametric statistics was chosen for the data analysis. 

A chi-square test was conducted to check that parents of toddlers (0–3 years) did not differ from those of pre-schoolers (4–5 years) in terms of gender, education, working status, lifestyle changes and variables related to the COVID-19 emergency. The same test was used to evaluate differences between toddlers and pre-schoolers in gender distribution.

Changes in parental sleep quality (PSQI total score) and habits (habitual bed and rise times) that occurred during the lockdown relative to the pre-pandemic period were assessed through Wilcoxon’s signed rank test. The same test was used to compare current and retrospective assessments of children’s sleep (CSHQ total score and sub-scores; bed- and rise times; frequency of naps). Data on children’s bed- and rise times have been extracted from the CSHQ and separately analyzed for weekdays and weekends, in light of previous literature showing weekday-to-weekend sleep/wake rhythm discrepancies in children [33,34,35]. The abovementioned analyses were conducted separately for the two age groups.

Differences between the two age groups in age (both parents’ and children’s), PSQI and CSHQ scores, as well as in sleep schedules, were analyzed by means of Mann-Whitney test, separately for current and retrospective assessments. Relationships between sleep quality (both parents’ and children’s), parents’ stress, lifestyle changes, mood and fear during the lockdown were investigated through Spearman’s analysis of correlation. 

Following statistical guidelines to correct for multiple testing without running a too high risk of Type II Error (see, for example, [36]), we applied an adapted Bonferroni procedure (as in [37,38]). The conventional alpha value (*p* ≤ 0.05) was divided by two, that is the number of relevant sleep “dimensions” addressed in our research (“sleep schedules” and “sleep quality”). Therefore, significance was set at *p* ≤ 0.025.

## 3. Results

### 3.1. Demographics

Sixty-one respondents (54.4% of the sample) reported having a child aged 0 to 3 years, while the remaining 51 participants (45.5% of the sample) had a child aged between 4 and 5. Demographic information about the sample is shown in Table 1. 

### 3.2. General Information and COVID-19 Items

Table 2 shows the distribution of the sample on general information items as well as COVID-19 items, separately for toddlers’ and pre-schoolers’ parents. The chi-squared test showed that the two groups of parents did not differ on any of the items.

### 3.3. Differences in Children’s Sleep Schedules across Time Points and Age Groups

**Figure 1:**
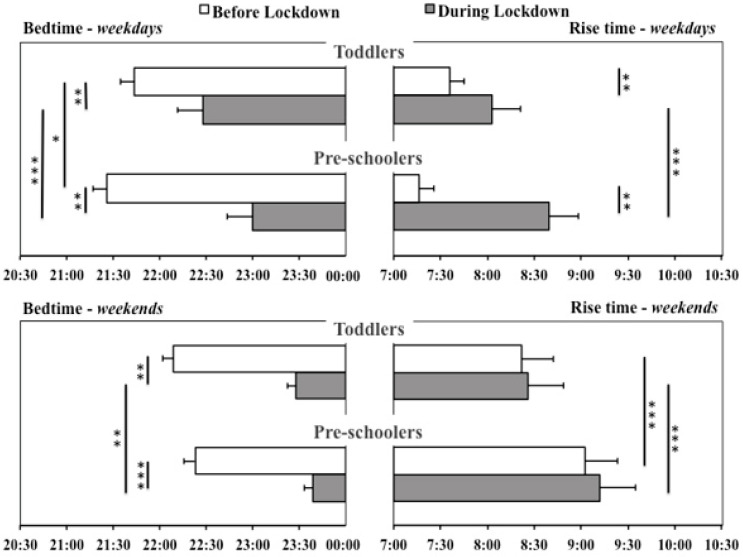
Bed- and rise times on weekdays and weekends in toddlers and pre-schoolers before and during the lockdown. Error bars represent standard error of the mean. Significant within-groups and between-groups comparisons are reported. * *p* < 0.025; ** *p* < 0.01; *** *p* < 0.001.

Figure 1 displays bed- and rise times (on weekdays and weekends) before and during the lockdown in toddlers and pre-schoolers. 

On weekdays, bed- and rise times were significantly delayed during the lockdown (compared to the pre-pandemic period) in both age groups (all four *p*’s < 0.003). This lockdown-related delay was more pronounced in pre-schoolers, as indexed by the fact that, relative to toddlers, their bedtimes were earlier before the lockdown (*p* = 0.020) and both their bed- and rise times were later during the lockdown (both *p*’s < 0.001).

As for weekends, bedtimes were delayed in both toddlers (*p* = 0.008) and pre-schoolers (*p* < 0.001) during the lockdown relative to before, whereas rise times were unchanged in both groups. Between-groups comparisons revealed that, before the lockdown, bedtimes did not differ between groups, whereas pre-schoolers went to bed significantly later during the lockdown (*p* = 0.008). Pre-schoolers also displayed later weekend rise times both before and during the lockdown (both *p*’s < 0.001).

Napping frequency increased during the lockdown in both toddlers (*p* = 0.004) and pre-schoolers (*p* = 0.01). Between-groups comparisons revealed that pre-schoolers napped more frequently than toddlers both before and during the lockdown (both *p*’s < 0.001). 

### 3.4. Differences in Children’s Sleep Quality across Time Points and Age Groups

**Figure 2:**
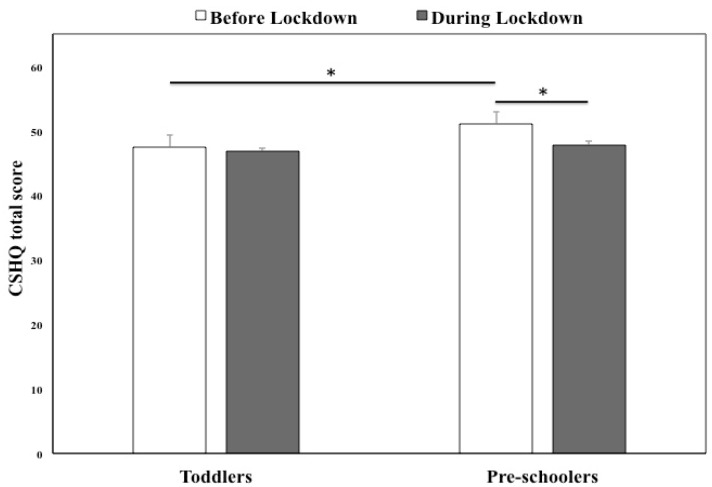
CSHQ total scores of toddlers and pre-schoolers before and during the lockdown. Error bars represent standard error of the mean. Significant within-groups and between-groups comparisons are reported. * *p* < 0.025.

Figure 2 displays CSHQ total scores before and during the lockdown in toddlers and pre-schoolers. Scores at the CSHQ subscales are shown in Table 3.

Toddlers and pre-schoolers showed different sleep quality profiles over time. While CSHQ total score was unchanged during the lockdown in toddlers, it was decreased in pre-schoolers (indicating improved sleep quality, *p* = 0.020). Accordingly, pre-schoolers showed significantly decreased scores at all the CSHQ subscales (all *p*’s < 0.025) except the Sleep Onset Delay and Sleep Disordered Breathing subscales, which were unchanged, and the Daytime Sleepiness subscale, which, instead, showed increased scores (*p* < 0.001). In toddlers, CSHQ scores were decreased only at the Bedtime Resistance, Sleep Onset Delay, Night Waking and Parasomnias subscales (all *p*’s < 0.01), whereas the Daytime Sleepiness subscale showed increased scores (*p* < 0.001). 

As for comparisons between toddlers and pre-schoolers, during the pre-pandemic period the two groups differed in several CSHQ indices. In fact, relative to toddlers, pre-schoolers displayed higher CSHQ total scores (*p* = 0.022), i.e., lower sleep quality, as well as higher scores at several subscales (Bedtime Resistance, Sleep Duration and Night Awakenings, all *p*’s < 0.025). Only the Daytime Sleepiness subscale showed the opposite trend, with toddlers scoring significantly higher (*p* = 0.001). Interestingly, all differences between groups (including both the CSHQ total score and its sub-scores) disappeared during the lockdown. 

### 3.5. Changes in Parents’ Sleep during the Lockdown

Adults’ sleep timing was markedly affected by the lockdown, with parents of both toddlers and pre-schoolers reporting significantly delayed bed- and rise times relative to before the lockdown (all four *p*’s < 0.001). Both groups of parents also displayed worsened sleep quality, as indexed by the significant increase of PSQI total scores from the pre-pandemic period to the lockdown (toddlers’ parents: *p* = 0.016; pre-schoolers’ parents: *p* < 0.001). Of note, during the lockdown average PSQI scores were raised beyond the cut-off for “poor” sleepers in both groups.

No differences in sleep timing or quality emerged between toddlers’ and pre-schoolers’ parents either before or during the lockdown. Parents’ sleep schedules and sleep quality before and during the lockdown are displayed in Table 4 (separately for toddlers’ and pre-schoolers’ parents).

### 3.6. Relationships between Children’s Sleep Features and Parents’ Sleep, Behavioural and Psychological Measures during the Lockdown

Correlations among sleep, behavioural (lifestyle changes) and psychological variables (stress, mood, fear) in the total sample are reported in Table 5.

A significant negative correlation emerged between parents’ and children’s sleep quality measures (PSQI and CSHQ total scores, respectively), indicating that higher sleep quality in children was associated to lower sleep quality in parents and vice versa. 

Furthermore, parent’s PSQI total scores during the lockdown were found to positively correlate with PSS-10 scores and item scores on mood and fear, with worse sleep quality corresponding to higher stress, lower mood and higher fear. Instead, children’s sleep quality (CSHQ total score) showed opposite associations with these variables. 

## 4. Discussion

This study addresses the impact of the lockdown imposed by the Italian government during the first wave of the COVID-19 pandemic (spring 2020) on children’s and their parents’ sleep schedules and quality. Specifically, we aimed to investigate children’s sleep through the CSHQ, which has not previously been used in Italian COVID-19-related studies, as well as to explore possible differential effects of the pandemic emergency on toddlers (aged 0–3 years) and pre-schoolers (aged 4–5 years).

Firstly, we observed significant changes in sleep/wake schedules in the overall sample of children, with delayed bed- and rise times in both toddlers and pre-schoolers during home confinement. These findings confirm extant literature showing a phase delay in children [14,15,16], adolescents [16,39] and adults [6,7,8] during the COVID-19 pandemic. As for weekday/weekend discrepancies in sleep schedules, which have not been assessed by previous pandemic studies [13,14,15], we found that the difference in sleep timing between weekdays and weekends was drastically reduced during the lockdown, especially in pre-school children. 

Moreover, our findings on parents’ sleep schedules and sleep quality confirm, although in a smaller sample, the now ample literature on pandemic-related sleep changes in the adult population. In fact, both toddlers’ and pre-schoolers’ parents displayed delayed sleep timing (as in, e.g., [6,7,8]) as well as an impoverishment of sleep quality (as in, e.g., [3,7,8,9]). Notably, in both groups of parents, average PSQI scores, which did not reach the cut-off for “poor sleepers” in the pre-pandemic period, were raised above it during the lockdown. Also, unsurprisingly, we found that poorer sleep quality during the lockdown was associated with higher perceived stress, worsened mood, and greater fear of contagion in the parents.

As for children’s sleep quality, we found a significant decrease, during the lockdown, of CSHQ total scores (indicating improved sleep quality) in pre-schoolers but not in toddlers, whose scores remained stable. Overall, this finding is in line with previous studies showing that children’s sleep quality was, on average, less affected than that of adults by the pandemic [10,14,15] or was even improved [13]. Also, it is coherent with Lim et al.’s observation [12] that, in their larger sample of 593 parents (of children aged 3 to 16 years), 50% perceived an improvement of their child’s sleep, 42% found it unchanged, and only 7% reported a worsening. Instead, the result is in contrast to other studies reporting a worsening of children’s sleep quality during the first wave of the pandemic [15,16,17,18,20]. Interestingly, the only other study observing a clear-cut improvement of children’s sleep quality was that by Liu et al. [13], who used the same instrument employed in our study (the CSHQ). This observation suggests that the inconsistent results obtained on children’s sleep quality during the pandemic may be somehow related to the different instruments used. In fact, besides studies relying on single item assessments [18,19], most studies on the topic [14,15,16,17] used the Sleep Disturbance Scale for Children (SDSC, [40]), which specifically targets sleep disorders, whereas the CSHQ addresses a broader range of sleep dimensions. Therefore, it may be hypothesized that, despite a pandemic-related increase in children’s sleep disturbances (expressed by higher SDSC total scores in the abovementioned studies), their overall sleep quality (assessed through the CSHQ in our study and in [13]) is improved.

We also observed that both groups of children experienced increased napping frequency and sleepiness (indexed by the Daytime Sleepiness subscale of the CSHQ) during the lockdown. These findings are compatible with recent studies showing that the COVID-19 pandemic induced significant changes in the quantity and nature of physical activity in children, with increased sedentary behaviors and use of electronic devices (e.g., [41]), which have been shown to be associated with increased daytime sleepiness in children [42,43]. In line with this hypothesis, the increased napping frequency may be consequent to the higher sleepiness, paired with the greater opportunity to sleep during the day. However, since we did not collect data on the timing and duration of naps, nor on the use of electronic devices, specific hypotheses on this set of findings remain speculative. 

Our findings also point to age-related differences as a further, complementary, explanation of the discrepant data on children’s sleep quality during the pandemic. Indeed, as mentioned above, pre-schoolers and toddlers displayed different patterns of sleep quality changes across the two time points (before and during the lockdown), with no change in toddlers and a significant improvement in pre-schoolers. Our between-groups comparisons revealed that, prior to the lockdown, pre-schoolers showed significantly worse overall sleep quality (higher CSHQ total scores) than toddlers, whereas this difference disappeared during home confinement. The profile observed for sleep quality is coherent with that which emerged from our between-groups comparisons on sleep schedules before and during the lockdown. Indeed, in the pre-pandemic period, pre-schoolers went to bed earlier than toddlers during the week, probably due to their kindergarten schedules. Instead, during the lockdown, we observed an inverted pattern, with pre-schoolers showing later bed- and rise times than toddlers both on weekdays and weekends. Therefore, our overall pattern of findings suggests that the pandemic and related lockdown has affected older children to a greater extent than younger ones. A plausible hypothesis is that daily routines were less affected by the COVID-19 emergency in toddlers compared to pre-schoolers’ families. In fact, it is likely that in many cases mothers of the youngest children were already spending most of their time at home (either not working or on maternity leave) when the lockdown was mandated, whereas the households of older children would have undergone greater modifications to every-day schedules. Indeed, the proportion of parents reporting a moderate to extreme degree of lifestyle change with the lockdown was slightly higher in the pre-schoolers than the toddlers group, as was the proportion of parents reporting changes in working status. However, these between-groups differences in lifestyle and working status changes were non-significant. Another hypothesis is that factors affecting sleep features may have different weights at the different ages. In toddlers, sleep characteristics may be mainly determined by developmental factors, accounting for the deep structural and organizational changes of sleep that occur in the first years of life [22,23,24,25]. The stronger effect of this internal, biological, drive would explain the greater stability of toddlers’ sleep patterns and quality in the face of the major lifestyle changes brought about by the pandemic. In pre-schoolers, instead, the drive of environmental factors would be stronger than that of developmental ones in determining sleep characteristics. This explanation is in line with results from Gruber and colleagues [39], who highlighted the beneficial effects of school closure due to the COVID-19 pandemic on sleep in a sample of adolescents. According to the authors [39], changes in students’ daily routines, with distance teaching starting later than usual, allowed a re-alignment of their naturally delayed sleep patterns with externally imposed sleep-wake schedules. In line with this idea, we have previously suggested that school schedules and school-day structure should be reorganized according to children’s chronopsychological variations during the day [44]. 

Finally, a peculiar result emerged from our correlation analysis. We observed a negative correlation between parent’s PSQI scores and children’s CSHQ total scores, indicating an inverse association between parents’ and children’s sleep quality. This result is at variance with data from two other studies assessing relationships between parents’ and children’s sleep quality during the pandemic: Cellini et al. [15] did not find any significant association (although note that their study was conducted on children of a different age range, i.e., 6–10 years), whereas Di Giorgio et al. [14] showed, in a similar sample (aged 2–5 years), a positive relationship between mothers’ and children’s sleep changes, which was mediated by the mothers’ working condition. Also, this finding appears counterintuitive, especially in light of previous studies reporting a positive association between sleep quality in mothers and their children [45,46,47], as well as a negative impact of parents’ distress on their own sleep and on that of their children [48,49,50]. However, as suggested by Liu et al. [13], it is plausible that the increase of stress, fear and negative mood, along with the more flexible parental work schedules, could have negatively impacted parents’ sleep and also triggered in them more responsive and protective behaviors, which in turn positively affected their children’s sleep quality. This explanation is actually supported by the negative correlations we found between CSHQ total scores and parents’ psychological measures (stress, mood, fear). Furthermore, it has been suggested that the widespread introduction of smart-working during the lockdown, along with the increased time spent by parents in child-caring, could represent long-term protective factors for young children’s sleep [20,51]. Specifically, parents’ practices related to their children’s sleep, such as controlling pre-bed routines or their use of electronic devices, could have been enhanced during home confinement, with beneficial effects on their children’s sleep [20,51]. However, data on these behaviors have not been collected in our study. Future research is required to better clarify the relationships between parents’ and children’s sleep, highlighting the other possible individual and family factors involved, both in standard conditions and in stressful conditions such as those characterizing the COVID-19 emergency. 

A few caveats impose caution in interpreting our results. First, our small sample size and the use of non-probabilistic sampling limit the generalizability of our findings. Indeed, since there was no compensation for participating in the study, the questionnaire might have attracted mostly respondents with sleep problems (although the improvement we observed in children’s sleep leads to exclude that this bias affected children’s results). Moreover, the majority of respondents were mothers, probably because they are the primary caregivers of children within the age range considered in our study. In the same vein, our two groups of children differed in gender distribution. However, note that gender disparities in the frequency of sleep problems seem to appear only since late adolescence [33,38,52,53]. 

Another constraint concerns our use of subjective measurements. Specifically, the possibility that results were biased by parents’ misperceptions of their children’s sleep [10] should be acknowledged, especially since the survey method, compared, e.g., to sleep diaries administered over longer time frames, requires a single, general assessment. A response bias linked to demand characteristics could also have derived from our use of retrospective questions regarding the period preceding the lockdown. Still, this methodological choice is deemed to be sufficiently reliable by some authors [54] and should be considered in light of the unpredictable instantiation of the lockdown, which did not allow for an a priori planning of research procedures. Unsurprisingly, in fact, surveys including retrospective questions on the pre-pandemic period have been repeatedly employed in sleep studies conducted during the pandemic (e.g. [7,8,55,56]).

Finally, our adoption of a non-conservative statistical correction in data analysis, described in the methods, should be taken into account in interpreting our data. This choice was made in order to avoid a high risk of incurring in a Type II Error, which could have prevented the detection of specific differences between the age groups [51]. 

## 5. Conclusions

Overall, our findings allow us to draw two main conclusions: (a) the COVID-19 pandemic, along with the relevant lifestyle changes brought about by the restrictive measures, affected sleep timing at younger ages in as much a remarkable way as in the adult population, in line with what has been observed in previous studies on sleep during the pandemic; (b) moreover, our results add to the existing literature by highlighting that, when it comes to sleep quality, the comparison across age groups reveals important differences: while adults’ sleep is dramatically worsened during the lockdown, toddlers’ sleep is basically unchanged and pre-schoolers’ sleep is even improved. This pattern of results points to an important modulating role of age on the extent to which sleep has suffered from the pandemic. As a research agenda, our findings point to the importance of investigating whether these age-related differences in the sensitivity of sleep to the global emergency would also extend to other kinds of severe stressful events and abrupt lifestyle disruptions, either in the short- or in the long-term.

Furthermore, our findings point to the importance of more carefully considering the instruments used in sleep research on younger populations. In fact, our results on children’s sleep quality are in line with those obtained in another study using the same survey instrument [13], whereas they are discrepant from those obtained on similar age groups using a different questionnaire (e.g., [14,17]). Future theoretical and methodological efforts aimed at better understanding strengths and limitations of each instrument are needed for a clearer description of sleep in specific age groups and, all the more, to quantify sleep changes under particularly stressful conditions such as the COVID-19 pandemic.

## Figures and Tables

**Table 1:** Demographic characteristics of the sample and their differences between groups.

	*n*	Parents’ Age	Parents’ Gender	Children’s Age	Children’s Gender
Toddlers	61	34.84 ± 5.21	7 M, 54 F	1.69 ± 0.98	44 M, 17 F
Pre-schoolers	51	39.24 ± 8.58	5 M, 46 F	4.75 ± 1.23	22 M, 29 F
tot	112	36.85 ± 7.26	12 M, 100 F	3.09 ± 1.88	66 M, 46 F
		U = 2043.00	χ^2^ = 0.81	U = 3111.00	χ^2^ = 9.65
		*p* = 0.004	ns	*p* < 0.001	*p* = 0.002

**Table 2:** Statistics of respondents related to general information and COVID-19 items. All values are expressed in %.

General Information		Parents of Toddlers	Parents of Pre-Schoolers	Chi-Squared Test
Education level	Junior High School	6.6%	7.8%	χ^2^ = 7.41, ns
	High school	23.0%	45.1%	
	Graduation	42.6%	33.3%	
	Post graduation	27.9%	13.7%	
Profession	Employed	54.1%	52.9%	χ^2^ = 3.15, ns
	Self-employed	29.5%	21.6%	
	Student	1.6%	0.0%	
	Unemployed	14.8%	25.5%	
Work continuation	Yes	23.0%	15.1%	χ^2^ = 3.04, ns
	No	44.3%	60.8%	
	From home	32.8%	23.5%	
Helps in the management of children	Yes	85.2%	82.4%	χ^2^ = 0.17, ns
	No	14.8%	16.6%	
**COVID-19 items**				
How is your current lifestyle?	More sedentary	72.1%	66.70%	χ^2^ = 3.53, ns
	As usual	21.3%	15.70%	
	More active	6.6%	17.60%	
How much has your lifestyle changed?	At all	1.6%	0%	χ^2^ = 2.87. ns
	A little	16.4%	7.80%	
	Moderately	36.1%	43.10%	
	Extremely	45.9%	49.00%	
Do you know someone who has resulted positive to COVID-19?	Yes	32.8%	39.20%	χ^2^ = 0.50, ns
	No	67.2%	60.80%	
Do you know someone who has deceased because of COVID-19?	Yes	11.5%	23.50%	χ^2^ = 2.86, ns
	No	88.50%	76.50%	
How is your mood in general?	Very positive	3.30%	2.00%	χ^2^ = 1.49, ns
	Moderately positive	27.90%	35.30%	
	Neutral	41.00%	31.40%	
	Moderately negative	26.20%	29.40%	
	Very negative	1.60%	2.00%	
How scared do you feel?	At all	9.80%	3.90%	χ^2^ = 1.46, ns
	Moderately	72.10%	76.50%	
	Extremely	18.80%	19.60%	

**Table 3:** CSHQ total score and sub-scores before and during the lockdown in toddlers and pre-schoolers.

		Pre-Lockdown	Lockdown
CSHQ total score	toddlers	47.51 ± 8.26	46.82 ± 8.10
	pre-schoolers	51.12 ± 6.36	47.82 ± 8.08
Bedtime Resistance	toddlers	6.26 ± 2.59	5.70 ± 2.75
	pre-schoolers	8.63 ± 2.50	6.73 ± 2.91
Sleep Onset Delay	toddlers	1.52 ± 0.54	1.23 ± 0.78
	pre-schoolers	1.63 ± 0.69	1.37 ± 0.80
Sleep Duration	toddlers	4.74 ± 1.55	4.56 ± 1.66
	pre-schoolers	5.31 ± 1.09	4.53 ± 1.81
Sleep Anxiety	toddlers	4.52 ± 2.05	4.57 ± 1.66
	pre-schoolers	5.31 ± 2.26	4.49 ± 2.33
Night Waking	toddlers	3.80 ±1.71	3.43 ± 1.82
	pre-schoolers	4.55 ± 1.54	3.69 ± 1.86
Parasomnias	toddlers	12.07 ± 1.50	11.59 ± 1.72
	pre-schoolers	12.37 ± 1.31	11.53 ± 1.86
Sleep Disordered Breathing	toddlers	5.59 ± 0.67	5.57 ± 0.69
	pre-schoolers	5.78 ± 0.50	5.71 ± 0.64
Daytime Sleepiness	toddlers	9.00 ± 2.82	10.16 ± 1.58
	pre-schoolers	7.53 ± 2.53	9.78 ± 2.02

**Table 4:** Sleep schedules and sleep quality before and during the lockdown in toddlers’ and pre-schoolers’ parents.

		Pre-Lockdown	Lockdown
Bedtime (hh:mm)	toddlers’ parents	22:39 ± 01:02	23:21 ± 01:09
	pre-schoolers’ parents	22:18 ± 00:58	23:33 ± 01:24
Rise time (hh:mm)	toddlers’ parents	07:20 ± 0:44	07:58 ± 0:43
	pre-schoolers’ parents	07:05 ± 0:41	08:14 ± 01:13
PSQI total score	toddlers’ parents	4.26 ± 1.83	5.45 ± 3.33
	pre-schoolers’ parents	4.02 ± 2.12	6.06 ± 3.39

**Table 5:** Spearman’s correlation between sleep, behavioural and psychological variables during the lockdown in the total sample. * *p* ≤ 0.01; ** *p* ≤ 0.001.

	1	2	3	4	5	6
1. PSQI	-					
2. CSHQ	−0.433 **	-				
3. PSS-10	0.597 **	−0.422 **	-			
4. Lifestyle change	0.188	−0.210	0.306 *	-		
5. Mood	0.392 **	−0.248 *	0.490 **	0.161	-	
6. Fear	0.277 *	−0.252 *	0.342 *	0.263 *	0.358 **	-

Notes. PSQI: higher PSQI scores correspond to lower sleep quality. CSHQ: higher CSHQ scores correspond to lower sleep quality. PSS-10: higher scores indicate higher levels of perceived stress. Lifestyle change: higher scores correspond to more changes in lifestyle. Mood: higher scores reflect a more negative mood. Fear: higher scores indicate higher perceived fear.

## Data Availability

The data that support the findings of this study are available from the corresponding author upon reasonable request.
